# The Prevalence of Very Frequent Physical Fighting among Boys and Girls in 27 Countries and Cities: Regional and Gender Differences

**DOI:** 10.1155/2013/215126

**Published:** 2013-06-27

**Authors:** Monica H. Swahn, Lindsay Gressard, Jane B. Palmier, Huang Yao, Melissa Haberlen

**Affiliations:** Institute of Public Health, Georgia State University, P.O. Box 3995, Atlanta, GA 30302-3995, USA

## Abstract

*Objective*. Using nationally representative data, this study examined the prevalence of very frequent physical fighting (≥12 times per year) among youth in 27 countries and cities. Frequent physical fighting has rarely been reported in the previous literature despite the implications for research and practice. *Methods*. Analyses were based on the Global School-based Student Health Survey (2003–2008) and the 2009 US Youth Risk Behavior Survey. Multinomial regression analyses were conducted to determine gender differences in frequent fighting. Countries were categorized into five regions (Sub-Saharan Africa, Central and South America, Asia, Eastern Mediterranean, and the United States), and one-way ANOVA tests were used to determine regional differences. *Results*. The prevalence of frequent fighting was highest in Zambia (7.7%) and lowest in Myanmar (0.5%). Gender differences were found in 20 countries, with boys being more likely to report frequent fighting than girls. The prevalence of frequent fighting varied by region (*F*(3,22) = 4.78, *P* = .01), with the Eastern Mediterranean having a significantly higher prevalence of frequent fighting than Asia (*P* < .01). *Conclusion*. The prevalence of frequent fighting varies by gender in many countries and varies across world regions. More cross-national research is needed to better understand the sociocultural context of frequent fighting and to inform youth violence prevention efforts.

## 1. Introduction

Youth violence is a major international public health concern [1, 2]. In nearly every region of the world, adolescents and young adults comprise the majority of violent death victims [3], resulting in significant losses in the world's most productive citizens. Indeed, global surveillance efforts estimate that an average of 565 youth between the ages of 10 and 29 years old are victims of homicide each day, with an additional 20–40 youth violence-related injuries occurring for each homicide [1]. Apart from the potential for death or serious injury, youth experiencing violence, namely, physical fighting, are more likely than their nonviolent counterparts to engage in further risk and violence-related behaviors and to suffer from a myriad of negative physical and emotional health outcomes [1, 4–6]. Considering the magnitude and severity of these consequences, a growing body of the literature has sought to better understand the prevalence and risk factors for youth involvement in physical fighting both in the USA and internationally [1, 2, 7–12]. Most recently, the trends and social correlates of physical fighting among youth in 30 countries were presented [13].

One facet of physical fighting among youth that remains underexamined, however, is the phenomenon of very frequent physical fighting. Although a recent US study indicates that frequent physical fighting (at least 12 times per year) is a relatively rare behavior among high school students, the well-being of those youth engaged in frequent fighting is of concern; adolescents reporting frequent physical fighting are at a heightened risk for suicide and other psychosocial problems when compared to students who engage in less frequent fighting or who do not fight [14]. Furthermore, adolescents who are frequent fighters may be more likely to become chronic offenders, a group that accounts for more than half of all the serious crimes committed by juveniles in the USA [15]. Stark gender differences in the prevalence of frequent fighting are also of note. Among US youth, 4% of boys versus 1% of girls report frequent physical fighting [14].

Internationally, comparative studies examining the occurrence of physical fighting among youth are nearly absent from the current literature, and even fewer studies have examined frequent fighting, most of which limit analyses to youth in Europe and North America [10, 12, 13]. A lack of nationally representative samples of youth has been particularly problematic in producing cross-national comparisons [10, 13]. Moreover, although some studies have been able to examine cross-national patterns of adolescent physical fighting, especially using the Health Behaviour in School-aged Children Survey (HBSC), the measure of frequent fighting has been restricted by survey designs with a maximum value of “3 or more” [13] or “4 or more” fights per year [10, 12]. Nevertheless, findings from these studies indicate that more frequent fighting is linked to an increased risk for both injury and additional risk behaviors across different countries and regions of the world [10, 12]. Prevention and intervention efforts targeted to this specific population may therefore have the potential to maximize limited resources, particularly in low income countries.

In order to better inform global youth violence prevention efforts, additional research examining the prevalence of frequent fighting is clearly needed, in particular in low and middle income countries. Both nationally representative data and surveys that enable participants to report a greater range of fighting frequency will allow for a more detailed examination of youth violence patterns across countries and world regions. Furthermore, considering the significant gender differences found in the prevalence of frequent fighting among US adolescents [14], the assessment of gender patterns in countries with differing cultural practices and gender norms may be particularly informative. Accordingly, the current study seeks to determine the prevalence of very frequent physical fighting (at least 12 times per year) among boys and girls in 27 countries and cities using the Global School-based Student Health Survey [16, 17] and the US Youth Risk Behavior Study [18]. The study will also examine the regional differences by categorizing the countries and cities into five world regions: Sub-Saharan Africa, Central and South America, Asia, Eastern Mediterranean, and the United States (data were not available from Europe). A better understanding of frequent fighting from large, representative cross-national comparisons will inform the development of violence prevention strategies and intervention programs.

## 2. Methods

The current study is based on data from the Global School-based Student Health Survey (GSHS) [16, 17]. The GSHS was developed and supported by the World Health Organization in collaboration with the United Nations Children's Fund, the United Nations Educational, Scientific, and Cultural Organization, the Joint United Nations Programme on HIV/AIDS, and with technical assistance from the Centers for Disease Control and Prevention. The goal of the GSHS is to provide data on health behaviors and relevant risk and protective factors among students across all regions served by the United Nations. Country-specific questionnaires, fact sheets, public-use data files, documentation, and reports are publicly available from the Centers for Disease Control and Prevention and the World Health Organization and have been described in more detail elsewhere [19]. Briefly, the GSHS is comprised of a self-report questionnaire, administered primarily to students aged 13 to 16 years old within the time period of 2003 to 2008. The survey uses a standardized scientific sample selection process, common school-based methodology, and a combination of core questionnaire modules, core-expanded questions, and country-specific questions.

This study conducted secondary analyses of the publicly available data files for 25 countries and 2 cities (see Table 1 for list). The 25 countries were selected because a complete nationally representative data file was publicly available. Two cities (Beijing, China and Dar es Salam, Tanzania) were also included to expand the regional comparisons. All selected countries and cities used a two-stage cluster sample design. The first stage selected schools with probability proportional to enrollment size, and the second stage randomly selected classrooms in participating schools. All students in selected classrooms were eligible to participate in the survey. The number of study participants and response rates for each country and the two cities are provided in Table 1.

As a means of comparison, this study also included information from the biannual, nationally representative Youth Risk Behavior Survey (YRBS) of high school students in the United States [18]. For this study, data from the 2009 YRBS (*N* = 16, 410) were analyzed. The US high school students voluntarily completed the anonymous, self-administered questionnaire in school following local parental permission procedures. All 9th through 12th grades students in public, Catholic, or other private schools in the 50 states and District of Columbia were included in the sampling frame [20]. Approval to conduct these analyses was obtained from the Georgia State University Institutional Review Board.

The measure of very frequent fighting was based on one survey question which asked students to report the number of times they had been involved in a physical fight during the past 12 months. Question wording in the GSHS and YRBS was equivalent. Response options reflected 8 levels ranging from 0 times to 12 or more times. To measure the prevalence of “any fighting,” the variable was coded dichotomously to indicate either no physical fights or one or more physical fights. To measure “very frequent physical fighting,” the variable was coded again to indicate no physical fights, 1–11 physical fights, or 12 or more fights. Multinomial regression analyses were conducted to determine gender differences in the frequency of fighting across the selected countries and cities, with girls used as the reference group.

To facilitate regional comparisons, the 27 countries and cities were divided into five world regions: Sub-Saharan Africa, Central and South America, Asia, Eastern Mediterranean, and the USA. Table 1 outlines the countries and cities that are included within each region and an overview of the sample characteristics. Although Trinidad and Tobago is considered to be within the continent of North America, it was included in the Central and South America region for the purposes of this study. One-way ANOVA and Tukey's HSD post hoc testing were then used to determine any significant regional differences in the prevalence of physical fighting and very frequent physical fighting. The USA was omitted from ANOVA and post hoc testing due to the lack of multiple data points in that region.

## 3. Results

The prevalence of any fighting and very frequent fighting for each country is presented in Table 2. The prevalence of any fighting among students ranged from 15.9% in Myanmar to 57.7% in Djibouti. Similarly, the prevalence of very frequent fighting varied across countries and cities ranging from 0.53% in Myanmar to 7.7% in Zambia. Statistically significant gender differences among students reporting any fighting were observed in 21 countries and 2 cities; no gender differences were found among students in Kenya, Uganda, Zambia, or Philippines (Table 2). Among students, boys were significantly more likely to report very frequent physical fighting than girls in 18 countries and 2 cities. Six countries and one city demonstrated a relatively strong likelihood for very frequent physical fighting among boys versus girls, with odds ratios ranging from 7.56 to 15.60 (Uruguay, Myanmar, Beijing, Jordan, Lebanon, Libya, and Morocco). Three countries (Trinidad and Tobago, United Arab Emirates, and USA) reported moderate odds ratios for boys versus girls, including the USA in which boys were 5.13 times more likely than girls to report engaging in very frequent fighting (95% CI: 3.46–7.60). Several other countries and one city noted significant, but less marked gender differences, with odds ratios ranging from 1.57 to 4.02 (Botswana, Kenya, Namibia, Swaziland, Tanzania, Argentina, Indonesia, Sri-Lanka, and Thailand). Seven countries did not demonstrate a significantly higher risk for very frequent fighting among boys versus girls (Ghana, Uganda, Zambia, Guyana, Philippines, Egypt, and Oman).

**Figure 1:**
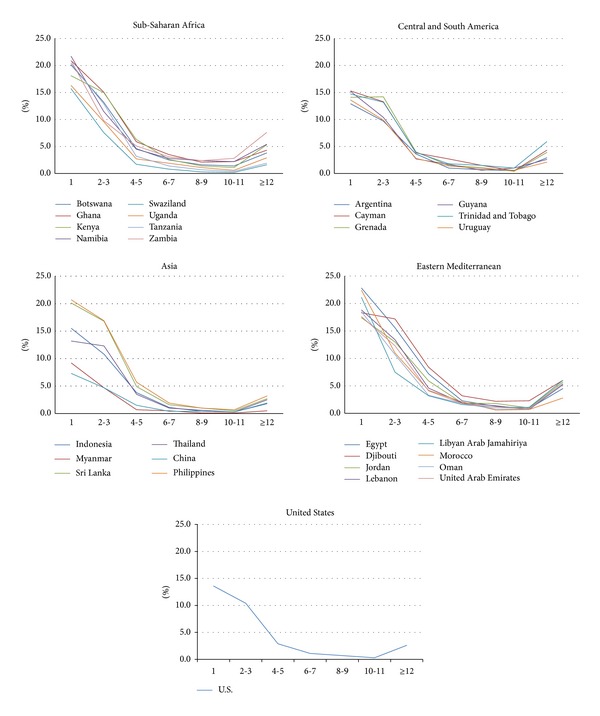
Distributions of the prevalence of fighting among students in selected countries, by region.

**Figure 2:**
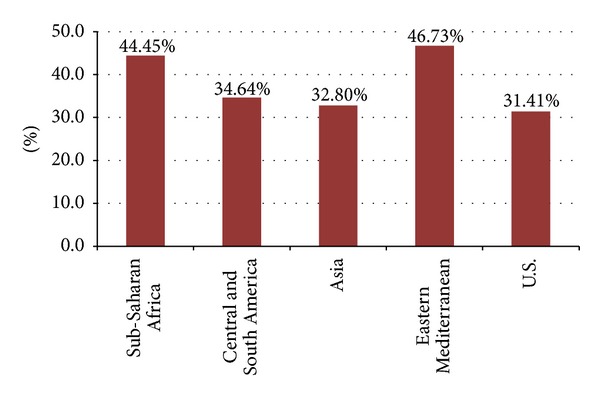
The Mean prevalence of any physical fighting (1 or more times) by region, GSHS and YRBS, 2009.

The distribution of fighting across the regions is presented in Figure 1. These distributions of the prevalence of student involvement in physical fighting by region are based on the number of times students reported fighting in the past year (1, 2-3, 4-5, 6-7, 8-9, 10-11, 12, or more). The mean prevalence of any fighting (one or more times) by region is presented in Figure 2. The Eastern Mediterranean region demonstrated the highest mean prevalence of any fighting (46.7%), while the USA had the lowest prevalence (31.4%). With the USA excluded, one-way ANOVA determined that the prevalence of any fighting varies significantly by region (*F*(3,22) = 3.43, *P* < .05), however, post hoc testing did not find significant differences between individual regions.

**Figure 3:**
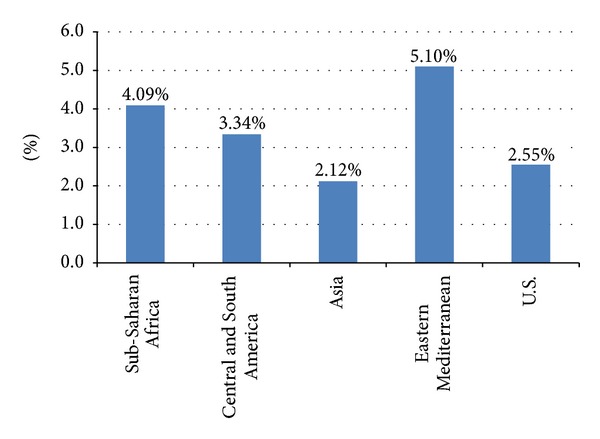
The mean prevalence of very frequent physical fighting (12 or more times) by region, GSHS and YRBS, 2009.

The mean prevalence of very frequent fighting (12 or more times) by region is presented in Figure 3. The Eastern Mediterranean region had the highest mean prevalence of very frequent fighting (5.1%), while the Asian region had the lowest prevalence (2.1%). With the U.S. excluded, one-way ANOVA determined that the prevalence of frequent fighting varies significantly by region (*F*(3, 22) = 4.78, *P* = .01). Tukey's HSD post hoc testing showed that the Eastern Mediterranean region has a significantly higher prevalence of frequent fighting than the Asian region (*P* < .01).

## 4. Discussion

This study examines cross-national population-based data on the prevalence of very frequent physical fighting across countries and regions of the world. Although involvement in any physical fighting is a common behavior, with over half of students reporting the behavior in some countries, the findings of this study indicate that very frequent physical fighting is a relatively rare behavior. Nevertheless, regional differences in the prevalence of very frequent fighting exist; countries representing the Eastern Mediterranean region exhibit a significantly higher prevalence of very frequent fighting than countries in the Asian region. As Smith-Khuri and Colleagues suggest [12], regional differences in the prevalence of frequent fighting indicate that frequent fighting may not simply be the product of “normal” developmental processes among adolescents. Instead, certain cultural norms and practices may either buffer against or contribute to the occurrence of very frequent fighting. Certain sociopolitical environments, such as political unrest or state of war, may also be a factor [2]. Further research should seek to determine those sociocultural factors that may affect youth reports of very frequent fighting and therefore provide a greater understanding to the associated risk factors. Providing a methodological example, a recent meta-analysis of cross-national research found that several sociocultural factors, such as age structure and income inequality, are significant predictors of criminal behavior, including homicide [21].

Supporting previous research [13, 14], the current study found that, in most countries selected for analyses, very frequent fighting was more likely to be reported by boys than girls. The magnitude of these gender differences, however, differed by country. The potential influence of gender norms on the report of very frequent fighting should thus be explored. Although the relationship context of the fighting reported in this study was not assessed, understanding the contribution of physical dating violence to the prevalence of frequent fighting may offer some important insight into the observed variation in the magnitude of gender differences. Previous cross-national research suggests that girls are particularly likely to fight within the context of intimate relationships, while boys more often fight with strangers [10]. Whether girls living in countries or cultures with stricter dating practices are less involved in frequent fighting may warrant exploration.

An additional aim of future research should be to determine whether higher rates of very frequent fighting among youth are associated with higher injury or death rates among young people in those countries. Previous cross-national research has found that youth reporting fighting four or more times a year are typically two to three times more likely than nonfighters to be hospitalized for an injury [10]. In this same study, the risk for injury among US youth was even greater; frequent fighters were more than 10 times likely than nonfighters to be hospitalized. While differences in health care access may be a factor, these findings suggest that cultural norms and practices may not only influence the frequency of fighting, but perhaps the severity of fighting or use of lethal weapons as well.

Interpretation of the findings in this report is subject to several limitations. First, all participants were school-attending youth, and as such, the findings may not reflect the experiences of youth who have dropped out of school or may not be able to attend school. Findings from a US study indicate that school attendance may be a protective factor for involvement in violence for both male and female students, thereby indicating a potential for underreporting of violent behaviors among school-attending youth [22]. The status of school attendance as a financial privilege in some countries may further affect these findings. Second, involvement in physical fighting may be considered a socially undesirable behavior, for girls in particular, thus potentially affecting the validity of self-report data. Third, the GSHS uses a single survey item to assess the frequency of physical fighting and, as such, does not assess the context of the fighting. The measure may or may not include incidents of fighting with siblings or other close relatives or friends in which there was no intent to harm. Fourth, comparisons across regions may be biased by the countries selected for inclusion, as well as the year for which data were collected. Lastly, the analyses and comparisons do not consider other demographic characteristics or societal level factors that may be relevant to frequent fighting. 

Despite these limitations, the findings of this study maintain important implications for expanding and improving future research on frequent fighting among youth. Of utmost importance is the need to establish uniform guidelines for assessing involvement in frequent fighting that would allow comparisons across populations and countries. Researchers currently use a broad range of classifications to assess frequent fighting (e.g., 4 or more times) [10, 12–14]. Validation studies are thus indicated to determine meaningful fighting frequency benchmarks that are associated with an increased risk for injury or other harmful outcome. Standardization of these benchmarks would aid epidemiologic research that may otherwise examine peer violence as a dichotomized construct, dividing youth into those who report no involvement in fighting versus those who report any involvement in fighting, thus ignoring the characteristics, circumstances, and precursors of frequent fighting. It is clear from this study and previous findings that focusing specifically on frequent fighting may elucidate important patterns and differences that may otherwise be missed when describing youth violence more broadly.

## 5. Conclusion

Given the variability in the prevalence of frequent fighting across countries and regions in this study, future research should examine the risk factors for fighting from an international and comparative perspective in order to inform prevention strategies that may have broader global relevance. Moreover, given recent cross-national comparisons outlining a decline in fighting across primarily European countries [13], research examining fighting trends in other countries and regions is also needed. Considerable gaps in research regarding the correlates and risk factors of frequent fighting specifically also remain. This study, which employed cross-sectional analyses of recent population-based surveillance data to understand the current prevalence and context of frequent fighting, provides insight for addressing these gaps. Furthermore, this study provides a starting point for further research to determine whether youth violence prevention interventions that specifically target frequent fighters present the opportunity for increased impact, particularly in areas with limited resource. 

## Figures and Tables

**Table 1:** Characteristics of Global School-based Student Health Surveys for selected countries and the US 2009 Youth Risk Behavior Survey.

Country	Year	Total sample	Type of representation	School response Rate	Student response rate	Participation rate	Boys (Wtd.%)	Girls (Wtd.%)
Sub-Saharan Africa								
Botswana	2005	2,197	National	100%	95%	95%	45.0%	55.0%
Ghana	2007	6,236	National	97%	86%	83%	53.4%	46.6%
Kenya	2003	3,691	National	96%	87%	84%	48.7%	51.3%
Namibia	2004	6,367	National	95%	86%	82%	45.2%	54.8%
Swaziland	2003	7,341	National	97%	99%	96%	36.2%	63.8%
Uganda	2003	3,215	National	90%	76%	69%	51.2%	48.8%
Tanzania	2006	2,176	Dar Es Salaam	100%	87%	87%	47.9%	52.1%
Zambia	2004	2,257	National	94%	75%	70%	48.9%	51.1%

Central and South America								
Argentina	2007	1,980	National	94%	82%	77%	48.0%	52.0%
Guyana	2004	1,212	National	100%	80%	80%	49.0%	51.0%
Trinidad and Tobago	2007	2,969	National	100%	78%	78%	49.8%	50.2%
Uruguay	2006	3,406	National	95%	75%	71%	45.2%	54.8%

ASIA								
Indonesia	2007	3,116	National	98%	95%	93%	49.9%	50.1%
Myanmar	2007	2,806	National	100%	95%	95%	50.8%	49.2%
Sri Lanka	2008	2,611	National	100%	89%	89%	50.0%	50.0%
Thailand	2008	2,767	National	100%	93%	93%	48.5%	51.5%
China	2003	2,348	Beijing	100%	99%	99%	50.5%	49.5%
Philippines	2003	7,338	National	99%	85%	84%	43.2%	56.8%

Eastern Mediterranean								
Egypt	2006	5,349	National	100%	87%	87%	51.9%	48.1%
Djibouti	2007	1,777	National	85%	98%	83%	60.2%	39.8%
Jordan	2004	2,457	National	100%	95%	95%	50.3%	49.7%
Lebanon	2005	5,115	National	92%	96%	88%	47.7%	52.3%
Libyan Arab Jamahiriya	2007	2,242	National	100%	98%	98%	50.1%	49.9%
Morocco	2006	2,670	National	100%	84%	84%	54.7%	45.3%
Oman	2005	2,979	National	100%	97%	97%	52.6%	47.4%
United Arab Emirates	2005	15,790	National	97%	91%	89%	50.0%	50.0%

United States								
US	2009	16,410	National	81%	88%	71%	47.8%	52.2%

**Table 2:** The prevalence of involvement in any and frequent physical fighting overall and by gender across 27 countries and cities.

Country	Prevalence of any fighting %			Prevalence of frequent fighting (>12) %			Multinomial logistic regression analysis of the association between sex and frequent fighting			
	Overall	Boys	Girls	Overall	Boys	Girls	Fought < 12		Fought ≥ 12	
							OR	OR (95% CI)*	OR	OR (95% CI)*
Sub-Saharan Africa										
Botswana	47.17	53.13	41.51	3.74	4.18	3.32	**1.60**	**1.30–1.97**	**1.57**	**1.03–2.39**
Ghana	53.50	52.18	55.01	4.31	4.55	4.04	**0.88**	**0.78–0.99**	1.06	0.78–1.43
Kenya	49.71	52.39	47.15	5.32	6.43	4.25	1.19	0.92–1.53	**1.68**	**1.08–2.61**
Namibia	50.26	55.18	46.19	5.32	6.43	4.40	**1.40**	**1.23–1.59**	**1.75**	**1.32–2.34**
Swaziland	27.99	37.58	22.62	1.65	2.97	0.92	**1.98**	**1.70–2.29**	**4.02**	**2.46–6.55**
Uganda	34.99	37.88	31.94	2.94	3.50	2.36	1.27	0.98–1.65	1.63	0.94–2.81
Tanzania	40.53	45.37	36.65	1.81	2.54	1.21	**1.40**	**1.17–1.68**	**2.43**	**1.09–5.40**
Zambia	51.45	49.28	54.01	7.66	6.29	9.28	0.87	0.64–1.18	0.61	0.37–1.03

Central and South America										
Argentina	31.27	43.83	19.63	2.93	4.36	1.61	**3.13**	**2.36–4.16**	**3.87**	**2.05–7.30**
Guyana	34.14	46.28	22.46	2.58	3.22	1.97	**3.03**	**2.33–3.95**	2.37	0.86–6.50
Trinidad and Tobago	41.84	55.89	27.88	5.81	8.90	2.75	**3.06**	**2.47–3.78**	**5.29**	**3.14–8.92**
Uruguay	31.32	46.09	19.16	2.05	3.70	0.69	**3.44**	**2.76–4.29**	**8.09**	**4.17–15.69**

ASIA										
Indonesia	33.76	46.95	20.63	1.83	2.28	1.39	**3.47**	**2.77–4.34**	**2.46**	**1.19–5.07**
Myanmar	15.86	21.71	9.80	0.53	0.98	0.07	**2.46**	**1.83–3.30**	**15.60**	**1.65–147.93**
Sri Lanka	47.28	60.27	34.34	2.69	3.26	2.12	**2.92**	**2.44–3.50**	**2.54**	**1.52–4.25**
Thailand	33.30	45.61	21.73	2.52	3.36	1.73	**3.04**	**2.41–3.84**	**2.80**	**1.56–5.04**
China	16.59	26.98	5.98	1.93	3.48	0.34	**5.36**	**3.74–7.70**	**13.10**	**4.58–37.47**
Philippines	50.02	51.63	48.80	3.22	3.59	2.94	1.11	0.92–1.34	1.29	0.86–1.95

Eastern Mediterranean										
Egypt	54.78	65.25	43.46	4.58	5.41	3.67	**2.45**	**1.65–3.62**	2.40	0.66–8.72
Djibouti	57.65	63.95	48.08	5.96	7.99	2.87	**1.78**	**1.45–2.19**	**4.01**	**2.56–6.28**
Jordan	45.91	64.09	27.55	5.33	8.39	2.24	**4.44**	**3.32–5.95**	**7.56**	**3.87–14.75**
Lebanon	45.98	64.56	28.99	5.24	8.92	1.87	**4.11**	**3.62–4.67**	**9.54**	**6.79–13.40**
Libyan Arab Jamahiriya	41.54	57.95	25.11	6.11	10.29	1.92	**3.66**	**2.82–4.76**	**9.52**	**5.66–16.03**
Morocco	43.69	62.18	21.31	2.78	4.39	0.82	**5.87**	**4.70–7.34**	**11.12**	**5.31–23.28**
Oman	41.10	45.73	35.97	5.31	5.75	4.83	**1.51**	**1.23–1.87**	1.40	0.91–2.18
United Arab Emirates	43.20	56.93	29.54	5.45	8.25	2.65	**2.96**	**2.64–3.32**	**5.09**	**4.02–6.44**

United States										
USA	31.41	39.26	22.90	2.55	3.99	0.99	**2.04**	**1.84–2.27**	**5.13**	**3.46–7.60**

*The multinomial logistic regression analyses predicted the odds for engaging in frequent and any fighting relative to not fighting, with girls as the reference group.

## References

[1] United States Department of Health and Human Services (2001). Youth violence. *A Report of the Surgeon General*.

[2] World Health Organization (2002). Youth violence. *World Report on Violence and Health*.

[3] Reza A, Mercy JA, Krug E (2001). Epidemiology of violent deaths in the world. *Injury Prevention*.

[4] Boynton-Jarrett R, Ryan LM, Berkman LF, Wright RJ (2008). Cumulative violence exposure and self-rated health: longitudinal study of adolescents in the United States. *Pediatrics*.

[5] Haynie DL, Petts RJ, Maimon D, Piquero AR (2009). Exposure to violence in adolescence and precocious role exits. *Journal of Youth and Adolescence*.

[6] Walsh S, Molcho M, Harel-Fisch Y (2010). Physical and emotional health problems experienced by youth engaged in violent behaviour. *Injury Prevention*.

[7] Craig W, Harel-Fisch Y, Fogel-Grinvald H (2009). A cross-national profile of bullying and victimization among adolescents in 40 countries. *International Journal of Public Health*.

[8] Harel-Fisch Y, Walsh SD, Fogel-Grinvald H (2011). Negative school perceptions and involvement in school bullying: a universal relationship across 40 countries. *Journal of Adolescence*.

[9] Pickett W, Schmid H, Boyce WF (2002). Multiple risk behavior and injury: an international analysis of young people. *Archives of Pediatrics and Adolescent Medicine*.

[10] Pickett W, Craig W, Harel Y (2005). Cross-national study of fighting and weapon carrying as determinants of adolescent injury. *Pediatrics*.

[11] Pickett W, Molcho M, Simpson K (2005). Cross national study of injury and social determinants in adolescents. *Injury Prevention*.

[12] Smith-Khuri E, Iachan R, Scheidt PC (2004). A cross-national study of violence-related behaviors in adolescents. *Archives of Pediatrics and Adolescent Medicine*.

[13] Pickett W, Molcho M, Elgar FJ (2013). Trends and socioeconomic correlates of adolescent physical fighting in 30 countries. *Pediatrics*.

[14] Swahn MH, Bossarte RM, Palmier JB, Yao H, Dulmen MHMV (2013). Psychosocial characteristics associated with frequent physical fighting: findings from the 2009 National Youth Risk Behavior Survey. *Injury Prevention*.

[15] Loeber R, Farrington DP (1999). *Serious and Violent Juvenile Offenders: Risk Factors and Successful Interventions*.

[16] Centers for Disease Control and Prevention Global School-based Student Health Survey (GSHS). http://www.cdc.gov/gshs/.

[17] World Health Organization Global School-based Student Health Survey (GSHS). http://www.who.int/chp/gshs/en/.

[18] Centers for Disease Control Youth Risk Behavior Surveillance System (YRBSS): Youth Online. http://apps.nccd.cdc.gov/youthonline/App/Default.aspx.

[19] World Health Organization Global School-based Student Health Survey (GSHS) purpose and methodology. http://www.who.int/chp/gshs/methodology/en/index.html.

[20] Brener ND, Kann L, Kinchen SA (2004). Methodology of the youth risk behavior surveillance system. *Morbidity and Mortality Weekly Report*.

[21] Nivette AE (2011). Cross-national predictors of crime: a meta-analysis. *Homicide Studies*.

[22] Blum J, Ireland M, Blum RW (2003). Gender differences in juvenile violence: a report from Add Health. *Journal of Adolescent Health*.

